# Metagenomic analysis of the effects of toll-like receptors on bacterial infection in the peritoneal cavity following cecum ligation and puncture in mice

**DOI:** 10.1371/journal.pone.0220398

**Published:** 2019-07-26

**Authors:** Pao-Jen Kuo, Cheng-Shyuan Rau, Shao-Chun Wu, Tsu-Hsiang Lu, Yi-Chan Wu, Peng-Chen Chien, Chia-Jung Wu, Chia-Wei Lin, Chia-Wen Tsai, Ching-Hua Hsieh, Chun-Ying Huang

**Affiliations:** 1 Division of Plastic Surgery, Department of Surgery, Kaohsiung Chang Gung Memorial Hospital and Chang Gung University College of Medicine, Kaohsiung, Taiwan; 2 Division of Neurosurgery, Department of Surgery, Kaohsiung Chang Gung Memorial Hospital and Chang Gung University College of Medicine, Kaohsiung, Taiwan; 3 Department of Anesthesiology, Kaohsiung Chang Gung Memorial Hospital and Chang Gung University College of Medicine, Kaohsiung, Taiwan; 4 Division of Trauma Surgery, Department of Surgery, Kaohsiung Chang Gung Memorial Hospital and Chang Gung University College of Medicine, Kaohsiung, Taiwan; University of the Pacific, UNITED STATES

## Abstract

**Objective:**

To establish the composition of bacteria in mice following cecum ligation and puncture (CLP) through metagenomic analysis and investigate the role of TLRs on the composition of bacteria.

**Methods:**

Total DNA extraction was done from the ascites, blood, and fecal samples from C57BL/6 mice sacrificed at 0, 4, 8, and 16 h, as well as from *Tlr2*^–/–^, *Tlr4*^–/–^, *Tlr5*^–/–^, and *NF-κB*^–/–^mice sacrificed at 16 h following CLP. Amplification of the V3–V4 regions of the bacterial 16S rRNA genes by PCR and the Illumina MiSeq sequencer was used for deep sequencing. Hierarchical clustering of the isolates was performed with Ward’s method using Euclidean distances. The relative abundance according to operational taxonomic unit (OTU) number or taxa was used to compare the richness among subgroups in the experiments.

**Results:**

There were 18 taxa that had significantly different abundances among the different samples of the C57BL/6 mice at 16 h following CLP. Various dynamic changes in the infectious bacteria inside the peritoneal cavity after CLP were found. While knockout of *Tlr5* and *NF-κB* impaired the ability of bacterial clearance inside the peritoneal cavity for some kinds of bacteria found in the C57BL/6 mice, the knockout of *Tlr4* enhanced clearance for other kinds of bacteria, and they presented excessive abundance in the peritoneal cavity despite their scarce abundance in the stool.

**Conclusion:**

NF-κB and TLRs are involved in bacterial clearance and in the expression pattern of the bacterial abundance inside the peritoneal cavity during polymicrobial infection.

## Introduction

The human gastrointestinal tract harbors a complex ecosystem [1, 2] comprised of an estimated 10^14^ microbes [3]. Currently, the mechanisms behind the immune tolerance of such a large microbial load are far from understood. The translocation of bacteria from the intestinal lumen into the peritoneal cavity had been proposed to be the basic mechanism for proceeding sepsis [4]. The bacteria that accumulate inside the peritoneal cavity are picked up by mesenteric lymph nodes, travel to the lungs and other organs, then trigger a systemic inflammatory response via the innate immune response, and eventually lead to the multiple organs dysfunction [4].

Toll-like receptors (TLRs) are highly conserved receptors of pattern recognition that function as key components involved in the innate defense against pathogens. Gut bacterial pathogens activate TLR signaling via the recognition of conserved microbial structures including lipopolysaccharide (LPS), lipoteichoic acid (LTA), and flagellin [5]. LPS is an integral part of gram negative bacterial wall and can be recognized by TLR4; LTA is an integral part of the cell wall of gram-positive bacteria and can be recognized by TLR2; flagellin can be recognized by TLR5 [6]. Activation of TLR leads to the massive release of inflammatory mediators into the bloodstream via the rapid transduction of signaling pathways, such as MAPK, NF-κB, and/or interferon responsive factors [7, 8]. Inflammatory cytokines such as tumor necrosis factor (TNF) can induce death receptor signaling with subsequent activation of inflammatory response [9, 10]. Signaling cascades that are crucial to the clearance of pathogens would be induced by the death receptors such as TNFR1, FAS, and TRAIL-R, which are also major targets for inhibition by these pathogens [11].

In the human gut, the order *Bacteroidales* are the major providers of LPS [12], which is a key mediator of the microbiome’s influence on host physiology and also one of the most potent activators of innate immune signaling [12]. Inhibition of TLR4 activation impairs bacterial clearance during sepsis [13]. The deletion of TLR4 in the intestinal epithelial cells leads to increased expression of mucin 2 [14]. The mucin 2 is secreted by goblet cells and is responsible for forming a mucus barrier that can change the composition and influence the growth of residual bacteria in the small intestine [15]. In addition, deletion of TLR2 leads to a significant change in the number and composition of the colonic mucosa-associated microbiome in *Tlr*2^-/-^ mice, with *Proteobacteria*, *Bacteroidetes*, and *Actinobacteria* being more abundant in the samples [16]. Nonetheless, although TLRs are deemed as major regulators of the host’s response to infections, it is unknown regarding that how the variability in TLR signaling may impact the growth or composition of bacteria during sepsis. Metagenomic analysis with next-generation sequencing has been used in recent years to identify the etiological agents of infectious diseases [17]. This method amplifies bacterial 16S rRNA genes with direct sequencing of millions of DNA/RNA molecules in a sample [18]; the pathogens can be inferred by matching the sequences to a database [19, 20]. Metagenomic analysis has also allowed the identification of previously uncharacterized bacteria that cannot live outside their hosts without symbionts [18] or is responsible for infectious diseases in the absence of bacterial cultivation [17, 21, 22]. In this study, we aimed to profile the bacterial composition in the ascites, blood, and fecal samples of mice following cecum ligation and puncture (CLP) using a metagenomic approach and investigate the role of TLRs on the composition of bacteria by using *Tlr* and *NF-κB* knockout mice.

## Materials and methods

### Animal experiment

C57BL/6 mice were purchased from the Taiwan National Laboratory Animal Center. *Tlr2*^*–/–*^(B6.129-Tlr2^tm1Kir^/J), *Tlr4*^*–/–*^(C57BL/10ScNJ), *Tlr5*^*–/–*^(B6.129S1-Tlr5^tm1Flv^/J), and *NF-κB*^*–/–*^(B6.Cg-Nfb1tm1Bal/J) mice were purchased from Jackson Laboratory (Bar Harbor, ME). Male mice aged 10–12 weeks and weighted 25–35 g were used in this study for CLP experiment to induce mid-grade sepsis according to the established protocol [23]. With the release of bacteria into the peritoneal space, CLP is considered to be the gold standard for creating a state of sepsis in animals [24]. Briefly, under the anesthesia with a combination of xylazine and ketamine, a midline abdominal incision was done. The mobilized cecum was ligated in the middle below the ileocecal valve and punctured once using a 21-G needle. A small amount of the bowel contents was extruded through the puncture holes to induce the polymicrobial peritonitis. The abdominal wall was closed in two layers. After surgery, the mice were resuscitated by subcutaneous injection of 37°C normal saline at 5 mL per 100 g body weight. Following national and institutional guidelines, the surgical procedures were performed and all housing conditions were maintained in an AAALAC-accredited, specific pathogen-free facility. The study was conducted after the protocol being approved by the Ethics Committee of Chang Gung Memorial Hospital Center for Laboratory Animals, with a project identification code 2016031001.

### Sample collection

The C57BL/6 mice were sacrificed at the indicated timepoints (4, 8, and 16 h) after the CLP experiments. Sham-operated mice that underwent the same procedure, including the opening of peritoneum and exposure of the bowel without ligation and needle perforation of the cecum, were used as control and were indicated as 0 h for comparison in this study. The *Tlr2*^*–/–*^, *Tlr4*^*–/–*^, *Tlr5*^*–/–*^, and *NF-κB*^*–/–*^mice were sacrificed at 0 h (sham-control) and 16 h after the CLP experiments. Three mice were used in each group in the experiments. During the indicated times, under anesthesia, 1 mL whole blood sample per mouse were drawn from the cardiac puncture and placed in tubes containing anticoagulant; the abdominal wall of the mice was open and, after 0.5 mL sterile normal saline was irrigated into the peritoneal cavity, 1 mL of ascites was drawn and collected; finally, the proximal cecum mucosa was incised and fecal samples were collected. According to the manufacturer’s protocol, DNA of the blood was extracted the collected samples using the QIAamp DNA Blood Mini Kit with a catalog no. 51104 (Qiagen, Hilden, Germany). The DNA of the ascites and stool were extracted by QIAamp DNA Mini Kit (catalog no. 51304) and QIAamp Fast DNA Stool Mini Kit (catalog no. 51604), respectively. The DNA was eluted with 400 μL of elution buffer and was then was isolated and purified. DNA concentration was determined using a Qubit 2.0 Fluorometer (Life Technologies, Invitrogen, CA)

### 16S metagenomic analysis

The V3–V4 region of the bacterial 16S rRNA gene was amplified by PCR using barcoded primers and fused with Illumina adapters which were overhang nucleotide sequences [25]. Two independent PCR reactions were done for each sample. According to the Illumina’s 16S Metagenomic Sequencing Library Preparation protocol (Illumina, San Diego, CA), these products were pooled and indexed. Sequencing and the processing of extracted data were performed at the Genomic and Proteomic Core Laboratory, Kaohsiung Chang Gung Memorial Hospital, where Illumina MiSeq platforms were utilized to sequence the samples. The Microbial Genomics Module of CLC Genomics Workbench 9.5.4 (Qiagen, Stockach, Germany) was used to analyzed the generated NGS data. The raw NGS reads were first trimmed in quality from the 3′ end, optimal merged with paired reads in fixed length trimming, and operational taxonomic units (OTUs) were clustered before taxonomic assignment as described [26]. The methods of trimmed mean of M-values (TMM) [27] was used for normalization of the data. In this method, the scaling factor is calculated by a weighted trimmed mean over the differences of the log-transformed gene-count fold-change between the sample and a reference, typically set as one of the samples in the study. To avoid spurious labelled taxonomic units, bacterial species with OTU number less than ten were arbitrarily neglected for further analysis [28]. In this study, the R statistical package version 3.3.3 [27] was used for data analysis. Ward’s method in Euclidean distances was performed for hierarchical clustering of the isolates. The relative abundance according to OTU number or taxa was used for comparison of richness among subgroups of experiments. The normality of the data was ascertained by the Kolmogorov-Smirnov test. Comparisons of numeric variables were performed using the Kruskal-Wallis test with Tukey HSD post hoc test to identify significantly different bacterial taxa among different groups. A two-sided *p*-values of < 0.05 indicated a statistical significance.

## Results

### Hierarchical clustering of the bacteria

**Fig 1:**
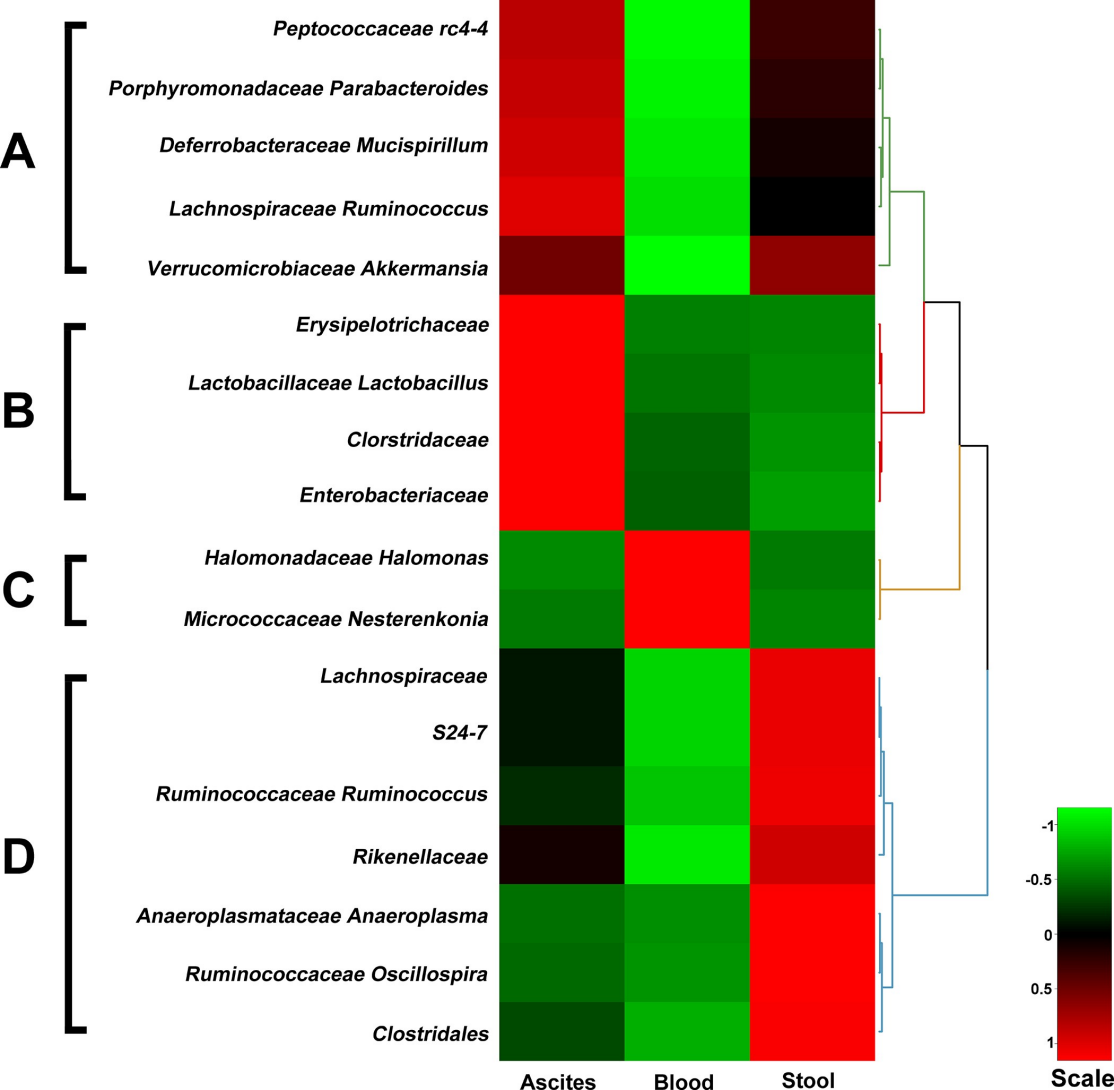
The microbiota composition of phyla in the samples of ascites, blood, and stool of the sham-operated C57BL/6 mice (0 h) and of the C57BL/6 mice receiving CLP for 4 h, 8 h, and 16 h.

**Fig 2:**
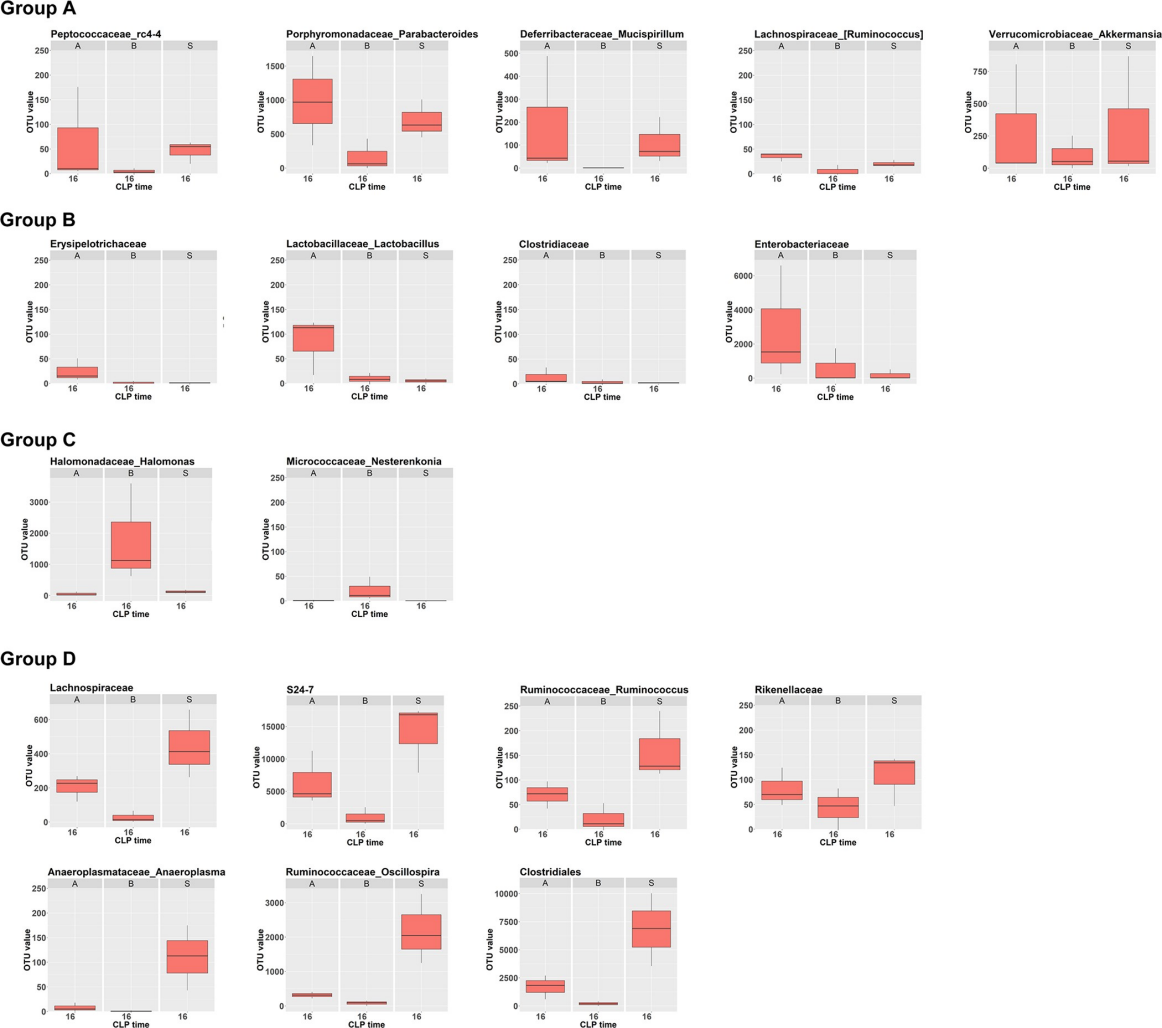
Hierarchical clustering of 18 bacterial taxa that had significantly different abundances among different samples of ascites, blood, and stool from the metagenomic analysis in the C57BL/6 mice.

**Fig 3:**
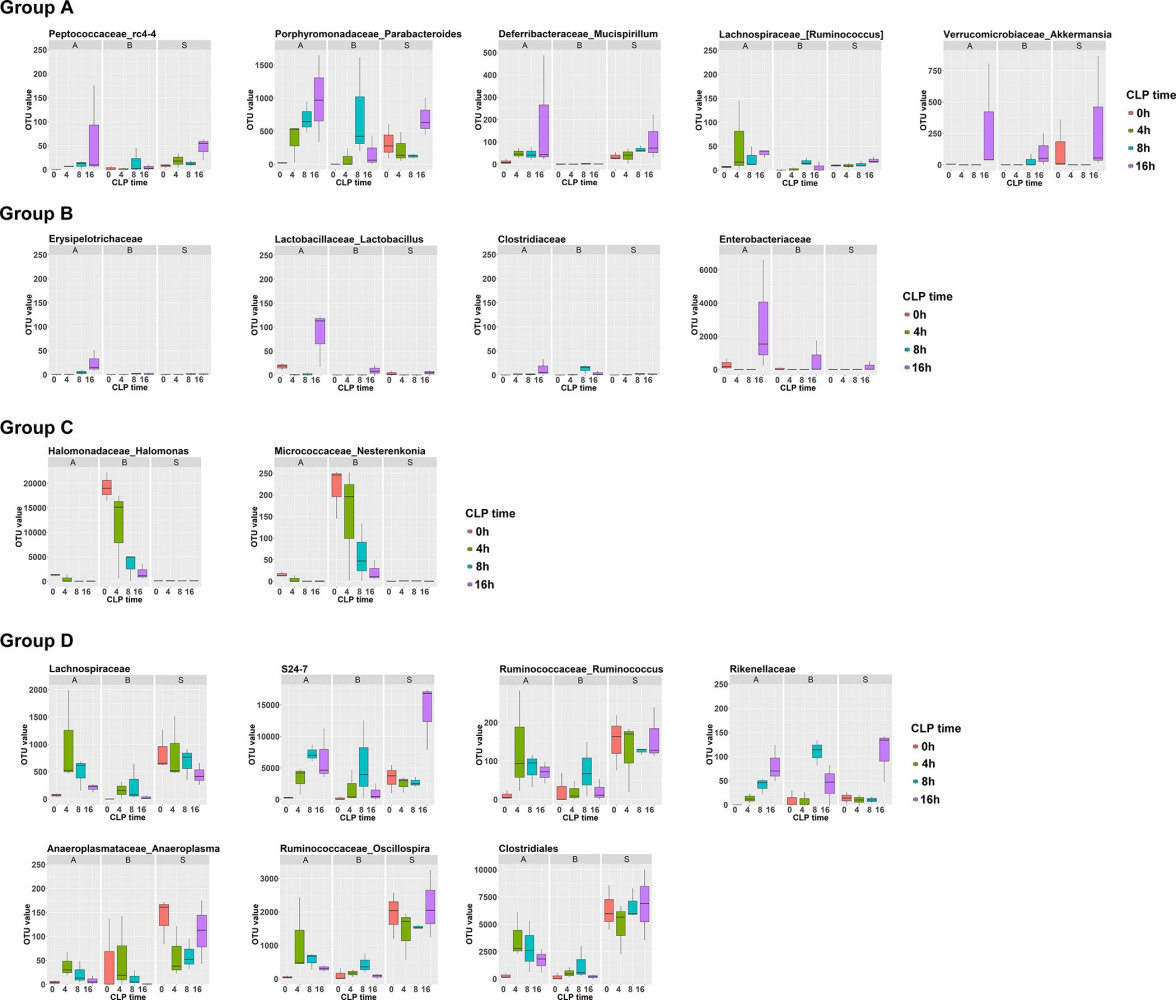
These bacteria can be divided into four groups according to their pattern of abundance across different samples at 16 h after cecum ligation and puncture (CLP) in C57BL/6 mice. Group A, the abundance of the bacterium between ascites and stool was similar but was much lower in the blood; Group B, the abundance of the bacterium in the ascites was much higher, regardless of a much lower abundance in the blood and stool; Group C, the abundance of the bacterium was much higher in the blood, but not in the ascites or stool; Group D, the abundance of the bacterium in the stool was high, but was lower in the ascites and blood. * indicated a significant different OUT number than those in the ascites.

From the metagenomic analysis, a total of 58 bacterial taxa were identified from the collected samples of C57BL/6 mice at 16 h after CLP experiment (S1 Table). The microbiota composition of ascites, blood, and stool was all dominated by three phyla: the Firmicutes, Proteobacteria, and Bacteroidetes (Fig 1). At the phylum level, the bacteria identified were predominantly members of *Firmicutes* (24) and *Proteobacteria* (23), followed by a much lower abundance of *Bacteriodetes* (5) and *Actinobacteria* (3), and a scarce abundance of *Deferribacteres* (1), *Tenericutes* (1), and *Verrucomicrobia* (1). Of these taxa, 18 had significantly different abundances across different samples (ascites, blood, or stool) and were subjected to hierarchical clustering (Fig 2). These bacteria can be divided into four groups according to their abundance pattern across different samples (Fig 3): Group A, the abundance of the bacterium was similar in ascites and stool but was much lower in the blood; Group B, the abundance of the bacterium was much higher in the ascites, regardless of a much lower abundance in the blood and stool; Group C, the abundance of the bacterium was much higher in the blood, but not in the ascites or stool; Group D, the abundance of the bacterium was high in the stool, but was lower in the ascites and blood.

### Time-dependent expression of bacterial 16s rRNA in C57BL/6 mice

**Fig 4:**
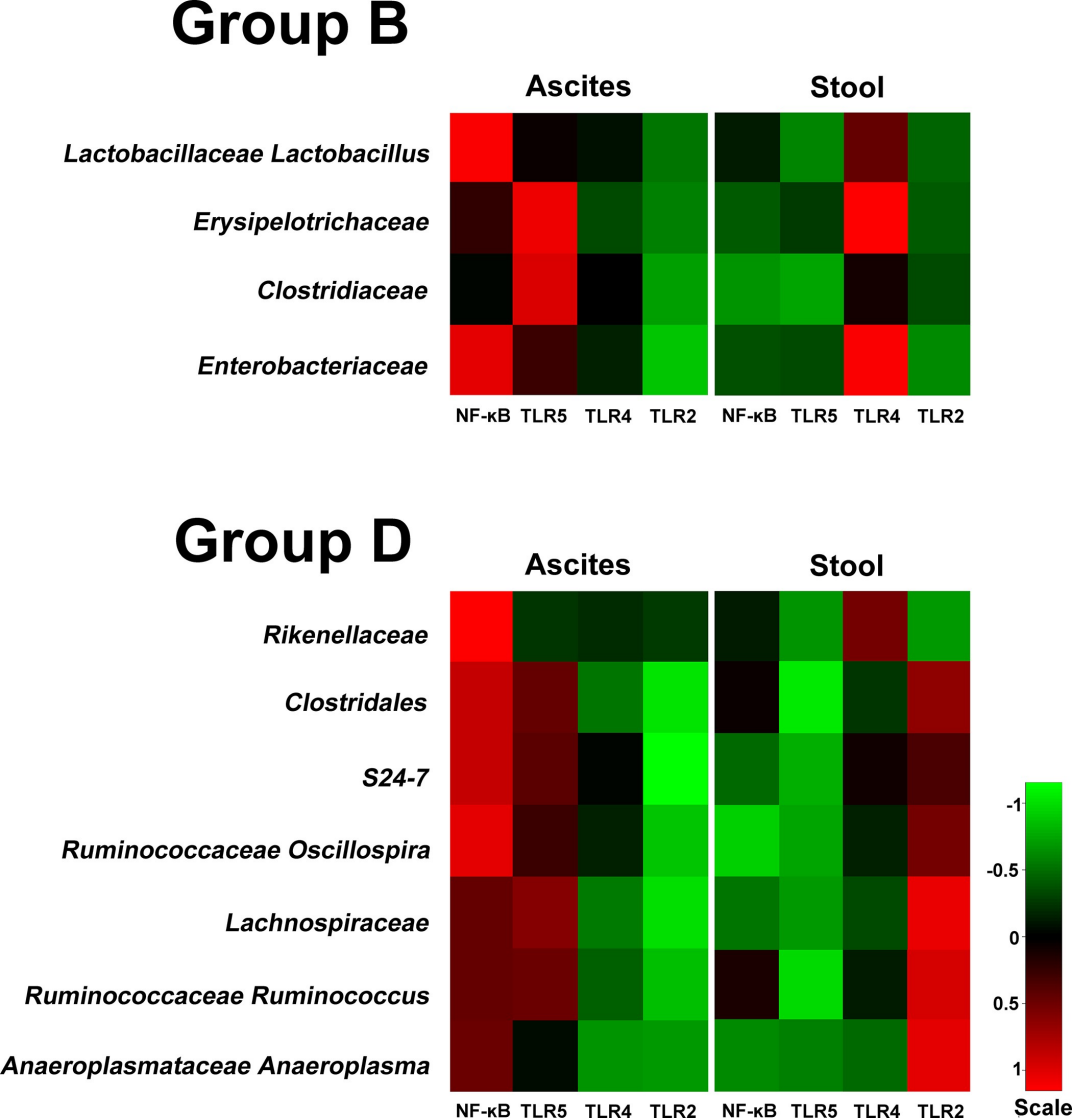
Time-dependent expression bacterial 16s rRNA from the metagenomic analysis of the isolated ascites, blood, and stool samples of C57BL/6 mice 0, 4, 8, and 16 h after CLP experiment. * indicated a significant different OUT number than those in the same sample (ascites, blood, or stool) from the sham-control C57BL/6 mice, which were indicated as 0 h.

The abundances of the selected 18 bacteria in the isolated samples of C57BL/6 mice 0, 4, 8, and 16 h after CLP experiment are shown in Fig 4; they reveal the dynamic change in the abundances of these bacteria during the process of polymicrobial infection. In Groups A and B, most of the bacteria had a time-dependent increase in abundance in the ascites after the CLP experiment. In contrast, in Groups C and D, following a presence or absence of a temporary increase at earlier times, a decrease in the abundance most of the bacteria (except *Rikenellaceae*) in ascites was observed at 16 h after CLP experiment. In the blood, a time-dependent decrease in bacterial abundance in Group C and a temporary increase with a following decrease in bacterial abundance in Group D were found.

### Expression of bacterial 16s rRNA in Tlr and NF-κB knockout mice

**Fig 5:**
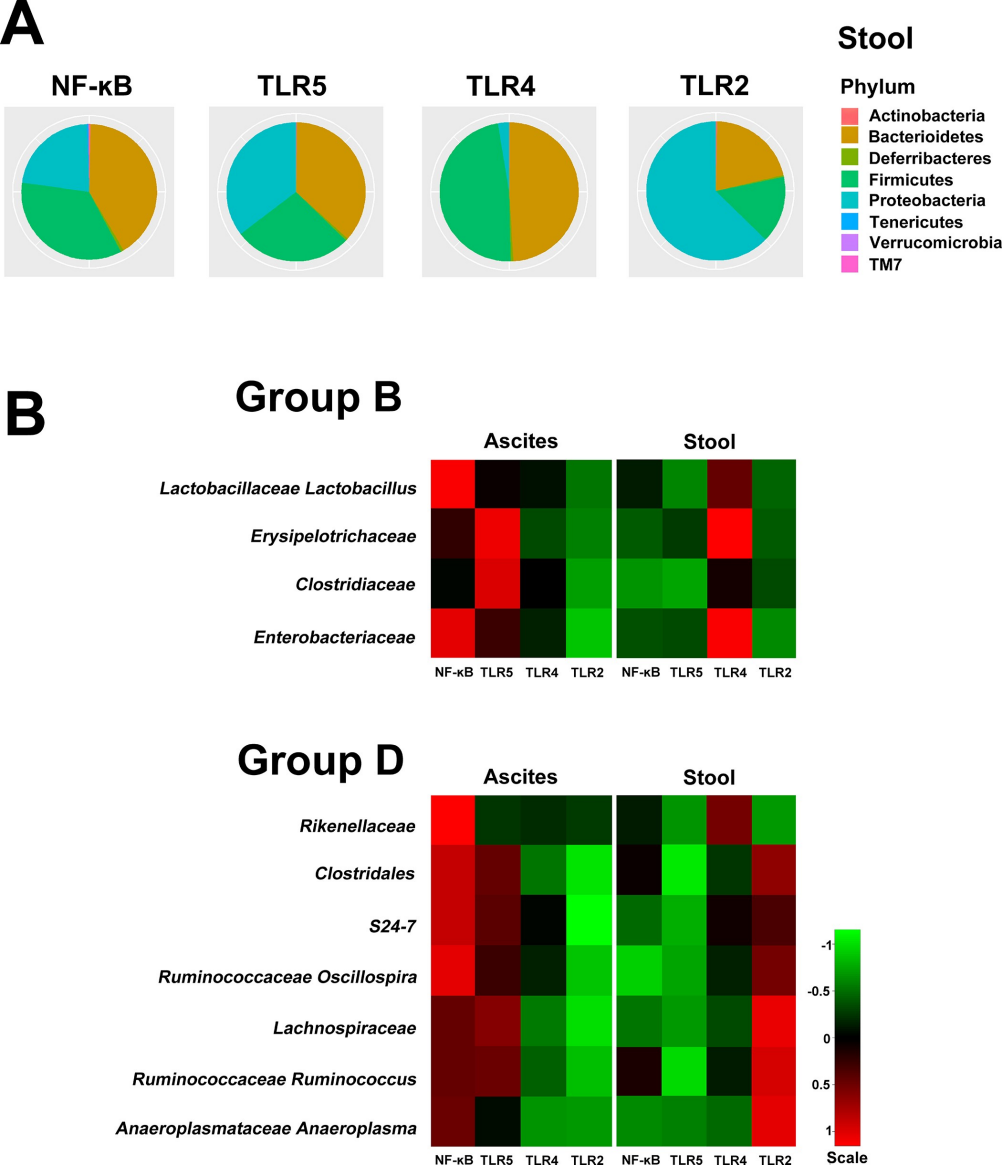
(A) The microbiota composition of phyla in the stool samples of the *Tlr2*^–/–^, *Tlr4*^–/–^, *Tlr5*^–/–^, and *NF-κB*^–/–^mice; (B), Hierarchical clustering of Groups B and C bacterial taxa from the ascites and stool samples from the metagenomic analysis in the *Tlr2*^–/–^, *Tlr4*^–/–^, *Tlr5*^–/–^, and *NF-κB*^–/–^mice. Quantity of 16S rRNA in the blood was not enough for the metagenomic analysis in the *Tlr* and *NF-κB* knockout mice.

The microbiota compositions of phyla in the stool samples of the *Tlr2*^–/–^, *Tlr4*^–/–^, *Tlr5*^–/–^, and *NF-κB*^–/–^mice were all dominated by three phyla: the *Firmicutes*, *Proteobacteria*, and *Bacteroidetes* (Fig 5A), which were similar with that identified from the stool of C57BL/6 mice. Of these four groups of bacteria, Groups B and D piqued our interest. The bacteria in Group B (*Lactobacillaceae*, *Erysipelotrichaceae*, *Clostridiaceae*, and *Enterobacteriaceae*) indicated failed bacterial clearance as there was an excessive abundance of these bacteria in the peritoneal cavity while they were scarce in abundance in the stool. In contrast, the bacteria in Group D (*Rikenellaceae*, *Clostridales*, *S24-7*, *Lachnospiraceae*, *Ruminococcaceae*, *Oscillospiraceae*, *Ruminococcaceae*, and *Anaeroplasmatoaceae*) indicated effective bacterial clearance as there was reduced abundance of these bacteria in the blood and in the peritoneal cavity, while the abundance of the bacteria was high in the stool. CLP experiments were performed in *Tlr* and *NF-κB* knockout mice to assess the null effect of these genes on the bacterial abundance in the isolated samples. However, the quantity of 16S rRNA in the blood of all *Tlr* and *NF-κB* knockout mice was not enough for metagenomic analysis, indicating that these bacteria were undetectable in the blood. In addition, expression levels of the 16S rRNA of the Group C and D bacteria in the ascites and stool of *Tlr* and *NF-κB* knockout mice at 16 h after CLP experiment are shown in Fig 5B. In *Tlr5*^*–/–*^and *NF-κB*^*–/–*^mice, the pattern of bacterial expression in Group B was not changed, which means that the abundance of bacteria in the ascites was much higher than that in the stool; however, the bacterial clearance for Group D bacteria was remarkably impaired. Although the bacterial load in the stool of *NF-κB*^*–/–*^and *Tlr5*^*–/–*^mice was lower than that in C57BL/6 mice, the bacterial abundance in the ascites was remarkably increased. This indicated that, in comparison with C57BL/6 mice, the knockout of *Tlr* and *NF-κB5* impaired the ability of bacterial clearance inside the peritoneal cavity for Group D bacteria. Such inhibition of bacterial clearance was not found in *Tlr4*^*–/–*^and *Tlr2*^*–/–*^mice, albeit the bacterial abundance of Group D in the stool of *Tlr2*^*–/–*^mice was higher. Notably, the pattern of 16S rRNA expression in Group B bacteria was changed in the *Tlr4*^*–/–*^mice, as there was effective bacterial clearance in the peritoneal cavity regardless of the abundant amount of these bacteria in the stool.

## Discussion

After CLP experiment, various dynamic changes in the abundance of infectious bacteria inside the peritoneal cavity were found. While knockout of *Tlr5* and *NF-κB* impaired the ability of bacterial clearance inside the peritoneal cavity for some kinds of bacteria found in the C57BL/6 mice, the knockout of *Tlr4* enhanced the bacterial clearance for other kinds of bacteria, which presented high abundance in the peritoneal cavity despite their scarce abundance in the stool.

In this study, Tlr5 and NF-κB were involved in the clearance of Group D bacteria, which include members of *Rikenellaceae*, *Clostridales*, *S24-7*, *Lachnospiraceae*, *Ruminococcaceae*, *Oscillospira*, *Ruminococcaceae*, and *Anaeroplasmatoaceae*. TLR5 contributes to antibacterial activity by targeting the flagellated bacteria of *Salmonella* [29] and *Pseudomonas aeruginosa* [30–32]. Activaiton of TLR5 initiates a signaling cascade and leads to the activation of NF-κB and subsequent proinflammatory pathways [29, 33]. Engagement of TLR5 with the resulting increased IL-1β are crucial for the clearance of bacteria by alveolar macrophages *in vitro* and *in vivo* [34]. TLR5 signaling plays an important role in protecting the lung [35] and liver [36] against circulating gut bacteria. Hepatocytes that lack TLR5 presented an impairment in the clearance of flagellated bacteria from the liver [36]. Administration of flagellin, a TLR5 agonist, restored the neutrophil response towards an N1 phenotype after burn injury and led to an increased clearance of bacteria inoculated in the woundbed [37]. In contrast, the absence of flagellin resulted in a slower clearance of the microorganism from the lungs and a delay time until death [38]. Furthermore, NF-κB plays a key role in the signaling pathways of host anti-microbial defences [11]. The inhibitor of kappa B (IκB) kinase (IKK) complex contains two catalytic subunits (IKKα and IKKβ), controls the activation of NF-κB transcription factors, dissociates NF-κB from IκB [39]. The NF-κB translocates into the nucleus and controls the transcription of inflammatory genes [39]. This proinflammatory response is followed by a compensatory immunosuppressive response that combines various functional impairment of immune cells [40], with the subsequently activation of signals that promote the clearance of bacterial infections [41]. Inactivation of IKKα in mice enhances inflammation and bacterial clearance [42]. In addition, the major importance of NF-κB in the pathophysiology of sepsis has been focused both in animals and in humans [43–45]. Deficiency of either cRel or p50, the subunits of NF-κB, results in impaired macrophages with impaired phagocytosis and decreased bacterial clearance, then increased mortality during sepsis [43, 44]. It has been shown that in the lungs of mice the activation of airway epithelial NF-κB promotes the clearance of *Mycoplasma pneumoniae* [46]; moreover, the mortality of mice with invasive *Streptococcus pneumoniae* was reduced via the NF-κB phosphorylation pathway by pretreatment with macrolides to significantly induce C-C motif chemokine ligand 2 (CCL2) in peritoneal macrophages [47].

According to our results, instead of effective bacterial clearance, bacterial infection in the peritoneal cavity was exaggerated for the bacteria in Group B (*Lactobacillaceae*, *Erysipelotrichaceae*, *Clostridiaceae*, and *Enterobacteriaceae*); however, the knockout of *Tlr4* enhanced bacterial clearance. Using hepatocytes and macrophages-specific knockout mouse strains to characterize the function of TLRs, studies demonstrated that one of the main functions of TLR4 on myeloid cells is to enhance phagocytosis and bacterial clearance [48]. In polymicrobial sepsis, the activation of TLR4 in macrophages is important for an effective bacterial phagocytosis [48, 49]. Absence of TLR4 on the hepatocytes actually enhanced clearance of bacteria during CLP [48]. The magnitude of the inflammatory response and the survival of the animals relied on the efficiency of bacterial clearance [48]. In addition, expression of TLR4 affects goblet cells or lysozyme secretion and alters the composition of microbiota [50]. Moreover, TLR4 expression in the intestinal epithelium coordinates the interaction between the luminal microbiota and genes which are important for metabolically pathways in the host [50], thus leads to changes of metabolic profile of the host [33, 50–53].

In this study, abundance of Group C bacteria (*Halomonadaceae* and *Micrococcaceae*) was found to be higher in the blood than in the ascites or stool. The members of the family *Halomonadaceae* typically occur in saline lakes, solar salt facilities, saline soils, and marine environments [54]; they have been reported to contaminate the bicarbonate used in preparing dialysis fluid and persist despite cleaning and flushing procedures [54, 55]. In addition, the family *Micrococcaceae* includes bacterial genera of gram-positive cocci that inhabit the skin and air [56]. A positive detection of bacteria from a sterile site such as blood can be due to either the translocation of bacteria or sample contamination during phlebotomy or sample handling [57]. The use of antiseptics to clean the venipuncture site may kill the bacteria, but does not destroy their DNA, and thus which could still be detected by the sequencing. Furthermore, commercially available DNA extraction kits may not be free of bacterial DNA [57]. Therefore, we speculate that the presence of these two bacteria may be attributed to contamination during the experiment.

In summary, we demonstrated that NF-κB and TLR are involved in bacterial clearance and correlate with the 16S rRNA expression pattern reflecting bacterial abundance inside the peritoneal cavity during the process of polymicrobial infection. However, the generalization of the results may be limited by the use of relatively few number of mice in this study and thus further investigation in the experiment with a larger scale of mice may provide more valuable information. Furthermore, there would be a bias in the bacterial profile once there was contamination in the study, albeit our animal center is an AAALAC-accredited specific pathogen-free facility.

## Supporting information

S1 Table Taxa of the bacteria identified from the collected samples of C57BL/6 mice at 16 h after CLP experiment. Bacterial species with OTU number less than ten were arbitrarily neglected.
(XLSX)
